# Crossmodal Effects in Task Switching: Modality Compatibility with Vocal and Pedal Responses

**DOI:** 10.5334/joc.129

**Published:** 2021-01-21

**Authors:** Denise Nadine Stephan, Johanna Josten, Erik Friedgen, Iring Koch

**Affiliations:** 1 Institute of Psychology, RWTH Aachen University, Aachen, Germany; 2 Institute for Automotive Engineering, RWTH Aachen University, Aachen, Germany

**Keywords:** task switching, cognitive control, modality influence, congruency effect, effect anticipation, compatibility, pedal responses

## Abstract

Modality compatibility refers to the similarity between the stimulus modality and the modality of response-related sensory consequences (e.g., vocal output produces audible effects). While previous studies found higher costs of task switching with stimulus-response modality-incompatible tasks (auditory-manual and visual-vocal), the present study was aimed to explore the generality of modality compatibility by examining a new response modality (pedal responses). Experiment 1 showed that the effect of modality compatibility generalizes to pedal responses when these replaced manual responses used in previous studies (i.e., higher switch costs when switching between auditory-pedal and visual-vocal tasks compared to switching between auditory-vocal and visual-pedal tasks). However, in single-task conditions there was no influence of modality compatibility. Experiment 2 was designed to examine whether modality compatibility depends on the frequency of task switches. To this end, one task occurred very frequently, overall decreasing the task switching frequency. Importantly, the results showed a robust task-switching benefit of modality-compatible mappings even for a highly frequent task, suggesting that the sustained representation of potentially competing response modalities affects task-switching performance independent from the actual frequency of the tasks. Together, the data suggest that modality compatibility is an emergent phenomenon arising in task-switching situations based on the necessity to maintain but at the same time separate competing modality mappings, which are characterized by ideomotor ‘‘backward’’ linkages between anticipated response effects and the stimuli that called for this response in the first place.

## INTRODUCTION

Performance costs arise almost always when more than one task has to be attended to at a time (e.g., Pashler, 2000, for a review). These costs of multitasking have been examined using various experimental paradigms, such as the dual-task paradigm or the task-switching paradigm (e.g. Koch, Poljac, Müller, & Kiesel, 2018, for a review). Specifically, in task switching, two or more different tasks are performed in a varying order, which typically leads to impaired performance on task-switch trials compared to task-repetition trials (switch costs; see, e.g., Kiesel, Steinhauser, Wendt, Falkenstein, Jost, Philipp & Koch, 2010; Monsell, 2003, for reviews).

Switch costs depend on a variety of factors, such as cue encoding benefits (e.g., Schneider & Logan, 2005; see also Jost, de Baene, Koch & Brass, 2013, for a review), preparation time (Meiran, 1996; Rogers & Monsell, 1995) or varied mapping of stimuli to tasks (e.g., Allport, Styles, & Hsieh, 1994; Koch & Allport, 2006; Waszak, Hommel & Allport, 2003). Importantly, even though a great diversity of influences on task switching has been examined (see also Vandierendonck, Liefooghe & Verbruggen, 2010), research on modality-specific influences is comparably rare. In fact, existing models of task switching do not incorporate mechanisms that could readily accommodate modality-specific influences. Therefore, the present study was aimed to examine and further specify the modality-specific influence on task switching based on the compatibility of stimulus modality and response modality across tasks (modality compatibility; see Stephan & Koch, 2010, 2011, 2016).

Stephan and Koch (2010, 2011, 2016) investigated the role of modality compatibility in task switching. They defined modality compatibility as “the similarity of stimulus modality and [the] modality of response-related sensory consequences” (Stephan & Koch, 2010, p. 1075). For example, vocal responses almost always produce auditory consequences, so that the modality of predictable and most salient response effects matches the modality of auditory stimuli, whereas vocal responses do not typically result in immediate visual consequences. Based on this modality-match consideration, auditory-vocal tasks are more modality compatible than visual-vocal tasks. Likewise, manual responses are typically more strongly associated with visible changes in the environment (like in eye-hand coordination) than with auditory effects, so that visual-manual tasks should be relatively more modality-compatible than auditory-manual tasks. In fact, the authors showed that switching between a visual-manual task and an auditory-vocal task (i.e., modality-compatible tasks) results in smaller switch costs compared to switching between visual-vocal tasks and auditory-manual tasks (i.e., modality-incompatible tasks), even though these modality-compatibility conditions did not differ across tasks in terms of the presented stimuli and the executed responses but only in terms of the modality mappings across tasks (see also Fintor, Stephan, & Koch, 2018). Similar modality-specific influences have also been found using dual-task paradigms (Hazeltine, Ruthruff & Remington, 2006; see also Huestegge & Hazeltine, 2011, for a review).

In order to explain the effect of modality compatibility, we argue that it arises from the anticipation of the sensory response effect (i.e., sensory feedback), based on the general concept of “ideomotor compatibility” proposed by Greenwald (e.g., 1972). The notion of ideomotor compatibility suggests that actions are controlled by the mental representation of their anticipated effects (for a review see e.g., Shin, Proctor, & Capaldi, 2010) and that a stimulus is compatible to a response to the degree of similarity between the stimulus and the anticipated response effect. For example, saying “one” in response to hearing “ONE” would be ideomotor compatible. In contrast, saying “X” in response to hearing “A” would not be ideomotor compatible because the stimulus does not exactly match the response effect in terms of semantic identity, but it would be modality compatible, which refers generally to the correspondence of stimulus and response-effect modality (i.e., both are auditory).

In comparison, the concept of ideomotor compatibility is rather narrow as it refers to the exact match between a stimulus and a response effect while modality compatibility refers to the general influence of the match between the stimulus modality and the modality of the anticipated sensory action effect. More specifically, we assume that the response-effect anticipation accompanying the selection and initiation of a certain response is modality-specific and thus induces activation of the associated task (i.e., the innate modality-mapping). In turn, this “ideomotor backward linkage” increases response-based between-task crosstalk and thus task confusion (e.g., Stephan & Koch, 2011). For example, the anticipation of the auditory feedback produced by vocal responses would prime processing of an auditory stimulus. While this process is beneficial in tasks with a modality compatible mapping, this response-based effect anticipation increases crosstalk between task sets in modality incompatible tasks (e.g., visual-vocal) because the anticipated sensory feedback (e.g., auditory) would prime processing the sensory stimulus referring to the competing task (i.e., auditory-manual). However, this mechanism is primarily relevant when simultaneous representation of competing stimulus and response modalities is required, for example to be able to switch between tasks, whereas it is much less relevant in single-task situations when only one stimulus and one response modality are relevant (see e.g., Stephan & Koch, 2010).^1^

Given that the crucial feature of modality compatibility is its higher generality compared to the narrower concept of ideomotor compatibility, it is important to examine whether the observed effects of modality compatibility in task switching are indeed generally due to the correspondence of the modality of the stimulus and the response effects rather than to some arbitrary features of the specific tasks used in the experiments. Previous experiments demonstrating the influence of modality compatibility on switch costs mainly used high spatial S-R compatibility across all modality mappings (Stephan & Koch, 2010). Importantly, the effect of modality compatibility on switch costs was also observed with arbitrary sets of stimuli and of responses that did not have any spatial dimensional overlap (see Kornblum, Hasbroucq & Osman, 1990; Stephan & Koch, 2011; see also Schacherer & Hazeltine, 2017). Replicating the modality compatibility effect with arbitrary S-R mappings suggests that S-R compatibility and the corresponding stimulus-based automatic response activation is not required for the effect of modality compatibility to occur.

Stephan and Koch (2016) already examined whether the influence of modality compatibility generalizes across different *stimulus modalities*. Stephan and Koch (2016) used tactile instead of visual stimulation, creating modality-compatible tactile-manual tasks and modality-incompatible tactile-vocal tasks. They demonstrated that the effect of modality compatibility on switch costs generalizes to tactile stimulation. However, regarding alternative *response modalities*, the issue of generality remains unclear. In a previous study, Stephan, Koch, Hendler and Huestegge (2013) replaced manual responses with eye movements, which should be most strongly coupled to the anticipation of visual effects. Against what was expected, using visual-occulomotor tasks instead of visual-manual tasks did not produce modality-compatibility effects on switch costs. The authors argued that eye movements may be special based on their involvement in the orienting reflex and that therefore visual orientation might be equally compatible with auditory and visual input. Yet, finding exceptions raises the issue of the generality of the rule.

The primary aim of the present study was to examine whether effects of modality compatibility in task switching can be generalized to other response modalities. To this end we used pedal responses instead of manual responses (see ***Table 1***). As argued above, we define the modality compatibility of a task regarding the functional characteristics of the specific motor response system. Specifically, for spoken responses, auditory stimuli are compatible as they lead to audible effects. Moreover, for pedal response visual stimuli should be modality compatible. Moving the feet is usually accommodated by intended locomotion, which in turn leads to visual changes in the environment, so that, according to our general definition of modality compatibility, visual-pedal tasks should be modality-compatible.

**Table 1:**
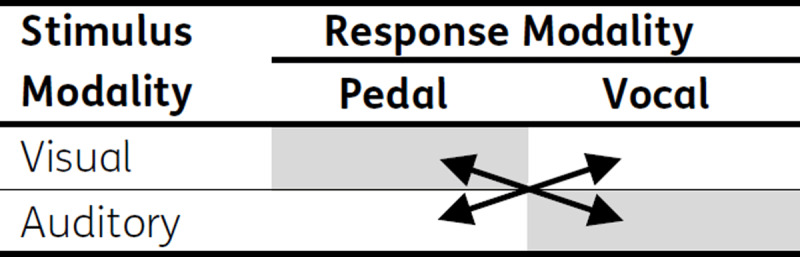
Stimulus and response modality combinations constitute modality compatibility. *Note*: Shaded cells indicate modality compatible mappings.

Previous studies found no influence of modality compatibility in single-task conditions, where there are no task switches. In the single-task condition, participants have absolute task certainty and do not need to keep both tasks active in working memory in order to flexibly switch from one task to another. In addition, the task sets decay over time and thus constantly switching between tasks might increase crosstalk. Thus the second aim of this study was to examine the influence of task frequency and switch recency on modality compatibility in task switching. We will further discuss the motivation for this experimental manipulation based on the results of Experiment 1.

## EXPERIMENT 1

Experiment 1 aimed at generalizing the results of Stephan and Koch (2010) by using a hitherto not investigated response modality. For this reason, the same experimental procedure was used as in Stephan and Koch (2010) but with pedal responses instead of manual responses (***Table 1***).

### METHOD

*Participants*. Sixteen participants with normal or corrected to normal vision and hearing acuity were tested (2 male, mean age = 24.6 years). The sample size was equal to that used in previous studies (Stephan & Koch, 2010, 2015), in which it was sufficient to detect the effect, which we wished to replicate with pedal responses.^2^

*Stimuli and apparatus*. The experimental procedure followed closely that of Stephan and Koch (2010), except for using pedal responses instead of manual responses (see ***Table 1***). The experiment was programmed in ERTS (BeriSoft Cooperation). Visual and auditory stimuli were used. Visual stimuli were white diamonds (1.5 × 1.5 cm) on a black screen presented either on the left or right side (1.25 cm distance from the center) of a 15-inch monitor (Multiscan 200 SX, Sony, Tokyo, Japan). Viewing distance was 60 cm. Auditory stimuli were 400 Hz tones presented via headphones (Speed Link SL 8755, Weertzen, Germany) either on the left or right ear.

Pedal and vocal responses were required. Pedal responses were given by pressing either the left or right switch (5 cm width × 4.7 cm height) on a slanting wooden board (44.4 cm width × 40 cm height) with the corresponding foot. The slope of the board was adjustable to the participants’ height. Vocal responses were the German words “links [left]” and “rechts [right]”. Speech onset was measured via voice key. Accuracy of vocal responses was coded online by the experimenter. Note that the S-R mappings were always spatially compatible.

*Procedure*. Written instructions were given to the participants on the screen at the beginning of the experiment. These instructions emphasized speed as well as accuracy. Participants were encouraged to ask the experimenter for further explanations if the task remained unclear.

**Figure 1:**
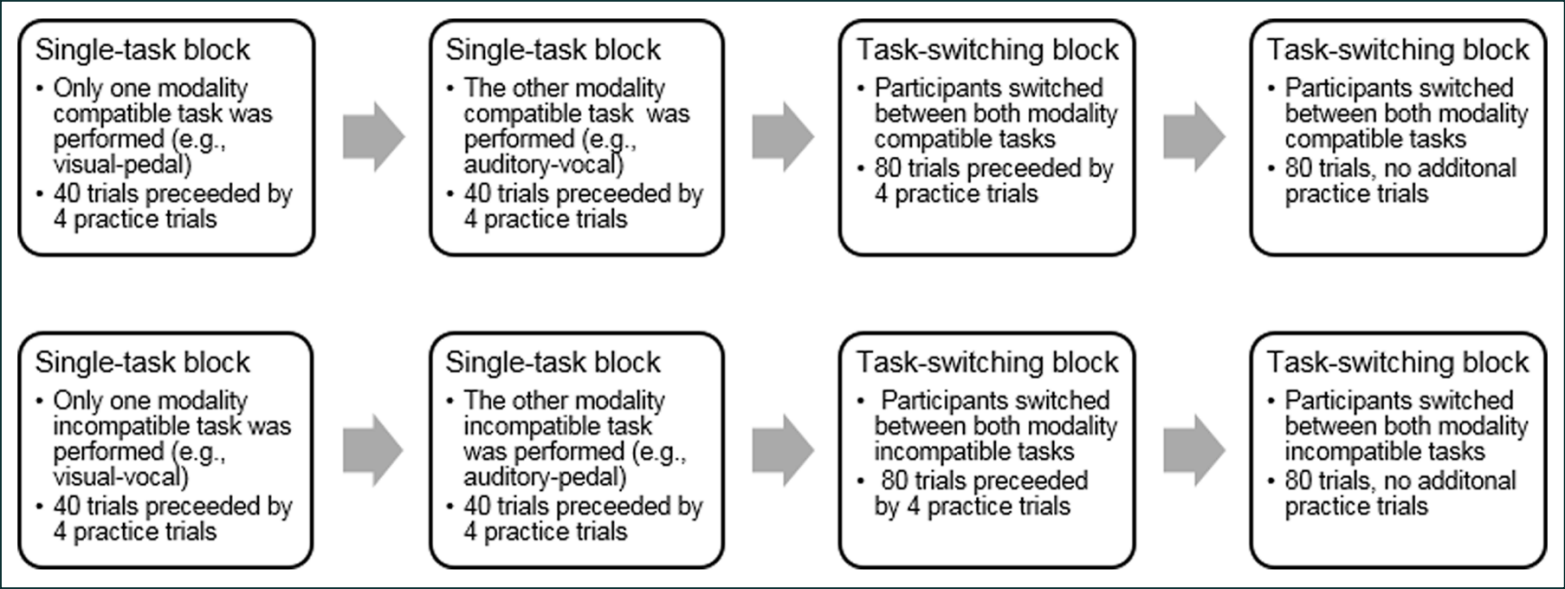
Order of Single-task blocks and Task-switching blocks in Experiment 1 depicted for participants starting with the compatible tasks (top) and for participants starting with the incompatible tasks (bottom).

Each participant received both the compatible and incompatible modality condition, with condition order counterbalanced across participants (see ***Figure 1***). Each condition consisted of two single-task blocks with 40 trials each and two task-switching blocks with 80 trials each. The single-task blocks included only one modality pairing each. The compatible single-task blocks were the visual-pedal and the auditory-vocal pairing. Incompatible single-task blocks were the visual-vocal and the auditory-pedal pairing. Prior to each single-task block, four practice trials were presented. The task-switching blocks combined either the two compatible or incompatible tasks of the previous single-task blocks. Four practice trials preceded the first task-switching block of each modality-compatibility condition (see ***Figure 1***). Task sequence within each block was randomised with the constraint that each task was presented equally often. Between each block, the participants could take a short break and were informed about their mean response time in the preceding block. The next block was initiated by the participant by pressing the spacebar.

Each trial started with the imperative stimulus. Stimuli (auditory and visual) were presented until the participants’ response or until 1,500 ms had elapsed without a response. The response-stimulus interval (RSI) in correct trials was 600 ms. In case of an error (i.e., if the wrong or no response was given), the German word “Fehler [error]” appeared on the screen for 500 ms. Thus, displaying the error feedback lengthened the RSI to 1,100 ms. The experiment lasted about 30 minutes.

*Design*. For the single-task blocks, the independent within-subject variable was modality compatibility (incompatible vs. compatible). For the task switching blocks, the independent within-subject variables were modality compatibility (incompatible vs. compatible) and task transition (switch vs. repeat). Response time (RT) and percentage error (PE) were the dependant variables.

### RESULTS AND DISCUSSION

The first two trials of each experimental block were excluded from analysis. The same applies to all responses given within the first 50 ms of each trial (0.7 %) due to being most likely voice-key artefacts. RTs were z-transformed for each subject within each experimental condition, and values not within the range of ±3 z were excluded from analysis (1.8 %). Furthermore, all error trials and trials following an error were discarded for RT analysis. After final publication, the data will be available on *https://osf.io/4q6pn/?view_only=9c3f52cfc43d471688f45422d73cacbd*.

Mean RTs and PEs were then collapsed across the compatible (visual-pedal & auditory-vocal) and across the incompatible (visual-vocal & auditory-pedal) condition. As input and output was identical in both modality-compatibility conditions, which differ only in the modality mapping across conditions, collapsing data across conditions eliminates specific effects based on stimulus modality or response modality alone (Hunt & Kingstone, 2004; Kreutzfeldt, Stephan, Sturm, Willmes, & Koch, 2015; Philipp & Koch, 2005; Sandhu & Dyson, 2012).

*Single-task analysis*: An analysis of variance (ANOVA) on single-task performance revealed no significant effect of modality compatibility for RT (incompatible: 368 ms vs. compatible: 376 ms), *F* < 1, and PE (incompatible: 2.9 % vs. compatible: 2.0 %), *F*(1,15) = 2.376, *p* = .144.

**Figure 2:**
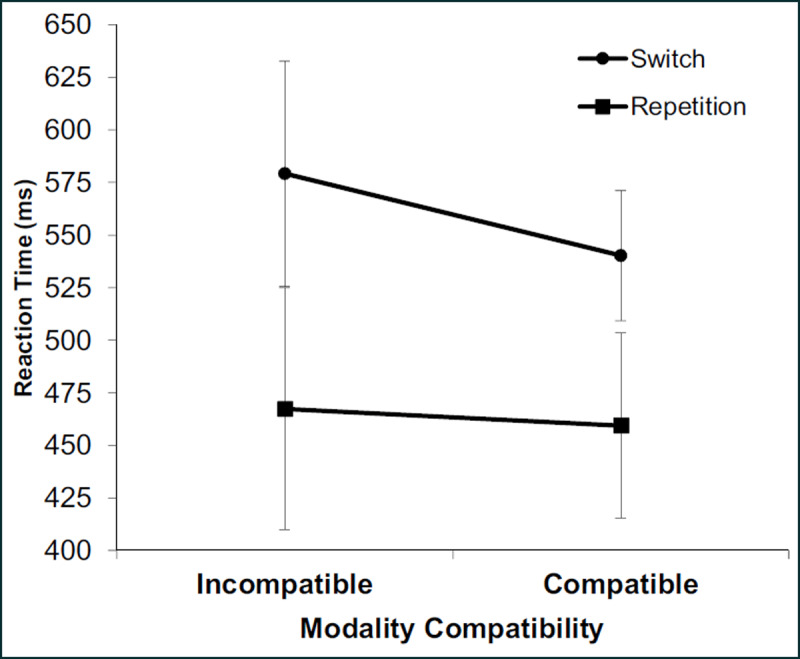
Mean reaction times (ms) in Experiment 1 as a function of modality compatibility and task transition. Error bars indicate standard deviation.

*RT task-switching analysis*: For the task-switching blocks, a two-way ANOVA on RT with task transition (switch vs. repetition) and modality compatibility (incompatible vs. compatible) as independent variables revealed significant main effects of transition, *F*(1,15) = 126.93, *p* < .001, η_p_^2^ = .894, indicating higher RT on task switches (560 ms) than on repetitions (463 ms). There was also an effect of modality compatibility, *F*(1,15) = 6.42, *p* < .05, η_p_^2^ = .300, indicating longer RT on incompatible (523 ms) compared to compatible modality mappings (500 ms). Importantly, the interaction between task transition and modality compatibility was also significant, *F*(1,15) = 19.68, *p* < .001, η_p_^2^ = .567, showing that incompatible modality mappings led to higher switch costs (112 ms) than compatible modality mappings (81 ms) (see ***Figure 2***).

Given that the main effect of modality compatibility was significant, we standardized switch costs for each condition based on the appropriate mean repetition RT to ascertain that higher switch costs with modality-compatible tasks were not just proportional to tasks with overall higher RT. An ANOVA for these proportional switch costs with modality compatibility (incompatible vs. compatible; 0.25 vs. 0.18) was significant, *F*(1,15) = 15.18, *p* < .01, η_p_^2^ = .503. Thus, the effect of modality compatibility on switch costs is not simply attributable to higher switch costs in incompatible tasks.

*PE task-switching analysis*: For PE, only the effect of transition was significant, *F*(1,15) = 7.76, *p* < .05, η_p_^2^ = .341, indicating higher PE on switch trials (7.9 %) than on repeat trials (4.9 %). The effect of modality compatibility, *F*(1,15) = 3.20, *p* = .094, as well as the interaction, *F*(1,15) = 1,00, *p* = .333, were not significant, even though they showed similar trends as the RT data (i.e, indicating higher PE on incompatible (7.4 %) compared to compatible modality mappings (5.5 %); higher switch costs on incompatible modality mappings (4.8%) than on compatible modality mappings (2.2 %) (see ***Table 2***).

**Table 2:** Mean % error and standard deviation (SD) for Experiment 1 as a function of modality compatibility and task transition.


	EXPERIMENT 1	

	INCOMPATIBLE	COMPATIBLE

	M (SD)	M (SD)

Switch	9.3 (7.6)	6.6 (4.5)

Repetition	5.5 (4.1)	4.4 (3.2)

Switch Costs	3.8	2.2


In summary, the data of Experiment 1 demonstrated that the modality-compatibility effect found with manual responses (Stephan & Koch, 2010, 2011, 2015, 2016) can be generalized to pedal responses. Note also that, like in previous studies, modality compatibility was beneficial only in task switching but not in single tasks. We account for this effect by assuming that the relative differences in modality compatibility are primarily relevant when a competing stimulus and response modality need to be represented simultaneously in working memory due to the requirement to be ready for an upcoming task switch. That is, increased interference and modality-specific crosstalk should occur mainly in task switching.

## EXPERIMENT 2

In Experiment 2, we investigated whether the effect of modality compatibility can still be observed in task switching when one of the tasks is presented only infrequently, revealing possible effects of task frequency and switch recency on modality compatibility. A manipulation of the frequency of one task, creating longer time between repetitions of this task, should lead to an increase in switch costs for incompatible modality pairings compared to compatible modality pairings.

The second goal concerns the basis of the modality-compatibility effect in task switching. Note that this effect was not found in single-task blocks, with absolute task certainty, in which no uncertainty about the correct stimulus or response modality exists. Experiment 2 was aimed to examine whether interference arising in task switching differs when the possibility of needing a different response modality is highly decreased. To this end, we manipulated task frequency to investigate whether the modality-compatibility effect is still present when one task is seldom and task switches are rare.

Earlier studies (e.g., Stephan & Koch, 2010, 2011) presented each task equally often in the task-switching blocks, so that in each trial, every task was to be performed with the same probability and task switches were frequent. Within this design, both tasks should hypothetically receive the same amount of activation as they are both equally probable and performed equally often throughout the experiment. In compatible modality pairings, the presentation of the stimulus in a certain modality and the corresponding response modality based on the modality of the anticipated response effects are assumed to automatically prime each other on every trial (Stephan & Koch, 2010, 2011), and the time between two task repetitions does presumably not influence this priming of the response modality. On the other hand, with incompatible modality pairings the instructed binding between stimulus and response modality is presumably weaker and priming takes place across task-sets and increases between-task crosstalk. However, it could be assumed that this crosstalk dissipates when the competing task has not been performed for a while and activation decays in working memory. Furthermore, it could be more difficult to inhibit the primed response modality in infrequent task-switch trials when it was already used in the frequent task-repetition trials beforehand. Therefore, if the frequency of tasks is manipulated, the performance difference between compatible and incompatible tasks might grow as a consequence of greater difficulty to select the correct response modality in the infrequently used incompatible task.

To test these hypotheses, in Experiment 2 we manipulated the frequency for one of two tasks in either modality-compatible or incompatible task-switching blocks and analysed the effects of task frequency on RT depending on modality compatibility. Note that Bonnin, Gaonac’h, and Bouquet (2011) found higher switch costs in blocks with a reduced probability for switches, yet these authors manipulated switch probability while holding task frequency constant. Moreover, we were specifically interested in a possible modulation of the influence of modality-compatibility on switch costs, not in an effect of frequency on overall switch costs.

Furthermore, we used tasks without spatial dimensional overlap (Kornblum, et al., 1990) to replicate the modality compatibility effect observed in Experiment 1 using different task characteristics (see also Schacherer & Hazeltine, 2017; Stephan & Koch, 2011). Thus, S-R mappings in Experiment 1 were defined by spatial S-R compatibility, so that this variable was kept constant across our manipulation of modality compatibility with pedal responses. In Experiment 2 we aimed to replicate the modality-compatibility effect with pedal responses using entirely arbitrary S-R mappings in order to avoid any influence of any stimulus-based automatic response activation that might arise with S-R ensembles that have dimensional overlap (Kornblum et al., 1990).

### METHOD

*Participants*. Sixteen new participants were tested (5 male, mean age = 23.4) and received either sweets or four Euro for participation. All had normal or corrected-to-normal vision and hearing acuity. (See Experiment 1 for the power analysis, since the sample size was the same.)

*Stimuli and apparatus*. The same modality pairings as in Experiment 1 were applied. Instead of spatial S-R mappings, arbitrary mappings without dimensional overlap between stimuli and responses were used (see Stephan & Koch, 2011). The spoken letters “*X*” and “*M*” served as auditory stimuli and were presented via headphones (Speed Link SL 8755, Weertzen, Germany). A white square and circle, each 1.5 cm wide and high, were used as visual stimuli and appeared in the middle of a 20 inch display (GDM-20E40T, ELSA, Germany). Vocal responses were given by saying “*A*” or “*Eins* [One]”. Pedal responses were given as described in Experiment 1. The experimental procedure was programmed in MATLAB (MathWorks). S-R mappings were counterbalanced across participants. Viewing distance was 60 cm.

*Procedure*. The instructions where the same as in Experiment 1. However, in contrast to Experiment 1, the experiment started with four single-task blocks. First were either two modality-compatible single-task blocks or two modality-incompatible single-task blocks containing 40 trials each. The order of modality compatibility as well as the order of both modality-compatible and incompatible single-task blocks was counterbalanced across participants. Before each block, participants were informed about which task would be presented (i.e. which stimulus and response modality were combined). Every block was preceded by four practice trials. After the practice trials, participants initiated the start of the experimental trials via keypress. Afterwards, four frequency-manipulated task-switching blocks of 160 trials each were presented. The participants were informed that one of the two tasks would appear less often than the other task but were not informed about the identity of the frequent task. Two of the four blocks included modality-compatible tasks and two blocks included modality-incompatible tasks. The task that was frequent in the first of the two blocks of the same modality compatibility was infrequent in the second block. The ratio of the frequent to the infrequent task was 7:1 (per block 140:20). In each block, direct repetitions of the rare task were presented exactly five times. Direct repetitions were necessary to allow calculation of switch costs for the infrequent task.

As in Experiment 1, vocal responses were coded online by the experimenter. Whenever participants made a mistake or did not answer within 2,500 ms, the word “Fehler [error]” appeared on the screen for 500 ms. If they did not speak loud enough for the voice-key to register, but an answer was coded by the experimenter, a message appeared to speak up (“Bitte lauter [louder please]”). The RSI was 600 ms for correct trials and 1,100 ms for incorrect trials. The experiment lasted about 30 minutes.

*Design*. For the single-task blocks, the independent within-subject variable was modality compatibility (incompatible vs. compatible). For the task switching blocks, the independent within-subject variables were modality compatibility (incompatible vs. compatible), task transition (switch vs. repeat), and task frequency (frequent vs. infrequent). The dependent variables were RT and PE.

### RESULTS AND DISCUSSION

Like in Experiment 1, RTs shorter than 50 ms (1.4 %) as well as the first two trials of each block were excluded from analysis. Z-scores were computed separately for each participant and each experimental condition to identify and exclude RT outliers (i.e., scores greater than ±3 z; 1.1 %). For RT analysis, all error trials (4.4 %) and trials directly following an error were excluded. Before final publication, the data will be available on *https://osf.io/4q6pn/?view_only=9c3f52cfc43d471688f45422d73cacbd*.

*Single-task analysis*: An ANOVA on single-task blocks revealed a significant effect of modality compatibility for RT, *F*(1,15) = 37.574, *p* < .001, η_p_^2^ = .715 (see ***Figure 2***), indicating higher RT in compatible (504 ms) than in incompatible conditions (438 ms). The ANOVA on PE revealed no significant effect of modality compatibility, *F*(1,15) = 1.526, *p* = .236. Note that the relative RT advantage for incompatible modality pairings in single-tasks is probably due to specifics of the arbitrary S-R combinations; importantly, this effect demonstrates that any beneficial effects of modality-compatible tasks in task switching may not be due to pre-existing differences in task difficulty because this would predict *smaller* switch costs for incompatible modality pairings.

*RT task-switching analysis*: For the task-switching blocks, we conducted a three-way ANOVA on RT with the independent variables transition (switch vs. repeat), modality compatibility (incompatible vs. compatible), and frequency (frequent vs. infrequent). It revealed significant main effects of transition, *F*(1,15) = 259.166, *p* < .001, η_p_^2^ = .945 (switch vs. repetition, 736 ms vs. 557 ms), modality compatibility, *F*(1,15) = 8.257, *p* < .05, η_p_^2^ = .355 (incompatible vs. compatible, 664 ms vs. 629 ms) as well as frequency, *F*(1,15) = 206.235, *p* < .001, η_p_^2^ = .932 (frequent vs. infrequent, 592 ms vs. 701 ms).

Importantly, the interaction between transition and modality compatibility was significant, *F*(1,15) = 40.599, *p* < .001, η_p_^2^ = .73, indicating higher switch costs in modality-incompatible tasks (222 ms) compared to modality-compatible tasks (136 ms). This result shows that the basic effect of modality compatibility in task switching is also present when task switches are rare and when the S-R mappings are arbitrary rather than spatially compatible across all modality mappings. To show that the effect of modality compatibility was not due to proportionally higher RT in switch trials in tasks with overall higher RT, the effect of modality compatibility was, like in Experiment 1, also analysed in terms of proportional switch costs, *F*(1,15) = 47.855, *p* < .001, η_p_^2^ = .761 (modality-incompatible vs. modality-compatible: 0.61 vs. 0.33), replicating previous findings.

**Figure 3:**
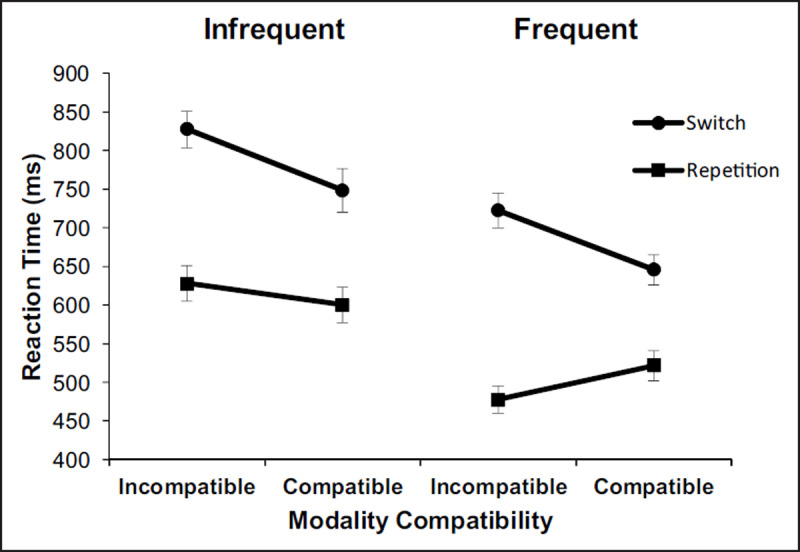
Mean reaction times (ms) in Experiment 2 for infrequent tasks (left panel) and for frequent tasks (right panel) as a function of modality compatibility and task transition. Error bars indicate standard deviation.

Furthermore, a significant interaction between frequency and modality compatibility was found, *F*(1,15) = 5.089, *p* < .05, η_p_^2^ = .253, showing a larger RT difference between infrequent modality compatible and incompatible tasks (54 ms) compared to the difference in frequent tasks (17 ms). The interaction between frequency and transition was not significant (*F* < 1). However, the three-way interaction between frequency, transition, and compatibility was significant, *F*(1,15) = 6.446, *p* < .05, η_p_^2^ = .301 (see ***Figure 3***).^3^

As this three-way interaction (in the RT data) shows, the modality-compatibility effect is modulated by frequency, as hypothesised beforehand. To follow up the significant three-way interaction between frequency, transition and modality compatibility, we conducted ANOVAs separately for the frequent and the infrequent tasks.

*Frequent tasks*: For frequent tasks, there was a main effect of transition, *F*(1,15) = 227.82, *p* < .001, η_p_^2^ = .938, indicating higher RT on switch trials (684 ms) than on repetition trials (499 ms). The interaction of transition and modality compatibility was significant, too, *F*(1,15) = 57.683, *p* < .001, η_p_^2^ = .183, showing larger switch costs in modality-incompatible (245 ms) than in modality-compatible (124 ms) conditions. The main effect of modality compatibility was just not significant, *F*(1,15) = 3.351, *p* = .087, η_p_^2^ = .794.

*Infrequent tasks*: The same analyses for infrequent tasks revealed a main effect of transition, *F*(1,15) = 167.779, *p* < .001, η_p_^2^ = .918, indicating higher RT on switch (788 ms) than on repetition trials (614 ms). The main effect of modality compatibility was significant, too, *F*(1,15) = 8.192, *p* < .05, η_p_^2^ = .353, indicating higher RT in modality-incompatible tasks (727 ms) compared to modality-compatible tasks (674 ms). Moreover, like for the frequent task, the interaction between transition and modality compatibility was significant, *F*(1,15) = 5.391, *p* < .05, η_p_^2^ = .264, revealing higher switch costs for modality-incompatible tasks (200 ms) compared to modality-compatible tasks (148 ms).

Thus, switch costs for modality-incompatible tasks were significantly higher in both frequency conditions. However, contrary to our hypothesis, the data showed a larger influence of modality compatibility on switch costs for frequent tasks than for infrequent tasks (121 ms vs. 52 ms).

*PE task-switching analysis*: For PE, the ANOVA using transition, modality compatibility, and frequency as independent variables yielded a significant main effect of transition, *F*(1,15) = 23.034, *p* < .001, η_p_^2^ = .606 (switch vs. repeat; 5.8 % vs. 2.2 %). The main effect of modality compatibility was significant, too, *F*(1,15) = 6.811, *p* < .05, η_p_^2^ = .312, indicating higher PE in modality-incompatible (4.8 %) than in modality-compatible (3.2 %) conditions. The interaction between transition and frequency was significant, *F*(1,15) = 6.628, *p* < .05, η_p_^2^ = .306, showing higher switch costs for infrequent tasks (5.2 %) compared to frequent tasks (1.9 %). The non-significant interaction between frequency and modality compatibility, *F*(1,15) = 4.221, *p* = .058, showed a trend towards a stronger modality-compatibility effect for infrequent (2.8 %) compared to frequent tasks (0.3 %). No other effects were significant, *F*(1,15) = 1.854, *p* = .193, for the interaction between transition and modality compatibility; all other effects: *F* < 1 (see ***Table 3***).

**Table 3:** Mean % error and standard deviation (SD) for Experiment 2 as a function of frequency, modality compatibility, and task transition.


	EXPERIMENT 2			

	INFREQUENT		FREQUENT	

	INCOMPATIBLE	COMPATIBLE	INCOMPATIBLE	COMPATIBLE

	M (SD)	M (SD)	M (SD)	M (SD)

Switch	8.6 (5.7)	5.1 (4.3)	5.4 (4.5)	4.0 (3.5)

Repetition	2.7 (4.8)	0.6 (2.5)	2.5 (1.1)	3.2 (2.0)

Switch Costs	5.9	4.1	2.9	0.8


Note that for frequent tasks, RT on task-repetition trials was similar to RT in single-task blocks, whereas a very distinct pattern was found for repetitions of the infrequent task, which might be due to a shift-bias expectancy (see ***Figure 2***). The absent benefit in repetition trials for infrequent tasks could be explained by the fact that only five trials in each block were repetition trials of the infrequent task and thus a rather rare event for participants. Since repetition trials in the infrequent modality-incompatible condition were not performed faster than in the modality-compatible condition, switch costs for the infrequent task were not increased to the same amount as for the frequent task condition, where a benefit for modality-incompatible tasks relative to modality-compatible tasks in repetition trials was found. Thus, whereas reduced reaction times can be observed for all tasks, the difference between frequency conditions is greatest for modality-incompatible repetition trials.

*Additional RT task-switching analysis –Switch recency*: In an additional exploratory analysis, we tested whether the number of successive repetitions of the frequent task had an effect on RT in switch trials of the infrequent task. To this end, we ran a 2x2 ANOVA with switch recency (one to four repetitions vs. five or more repetitions of the frequent task before a switch trial; 3122 vs. 4384 observations) and modality compatibility (compatible vs. incompatible) for the infrequent task. The main effect of switch recency was significant, *F*(1,15) = 10.763, *p* < .01, η_p_^2^ = .418, indicating higher RT in switch trials after five and more repetitions (807 ms) compared to fewer preceding repetitions (767 ms). The main effect of modality compatibility was significant as well, *F*(1,15) = 9.791, *p* < .01, η_p_^2^ = .395, showing higher RT on modality-incompatible switch trials (823 ms) than on modality-compatible switch trials (750 ms). The interaction between switch recency and modality compatibility was non-significant, *F*(1,15) = 3.331, *p* = .088. This result indicates, contrary to previous assumptions, that the incompatible bindings between modalities do not dissipate when the task is not performed over longer intervals. Again, due to comparatively low error rates, we did not calculate this analysis involving switch recency for PE.

In conclusion, Experiment 2 provided evidence that the effect of modality compatibility in task switching using pedal responses can also be observed using arbitrary S-R mappings (see also Stephan & Koch, 2011). Importantly, Experiment 2 showed, that the effect of modality compatibility on switch costs does not depend on high task and switch frequencies (i.e., how likely a task switch is). What matters is presumably the knowledge of having to perform another task with different modality pairings, even if this happens rather infrequently. Contrary to our hypothesis, switch-cost differences between modality-compatible and modality-incompatible tasks were even larger for frequent than for infrequent tasks due to differential RT in repetition trials. Nonetheless, the interaction between modality compatibility and transition was not only significant for frequent tasks but also for infrequent tasks and thus represents a very robust finding.

## GENERAL DISCUSSION

The aim of this study was two-fold to achieve a better understanding of the effect of modality compatibility in task switching. On the one hand we studied whether the effect of modality compatibility generalizes to other response modalities by using a hitherto not investigated response modality. To this end, we used pedal responses instead of previously used manual responses (Stephan & Koch, 2010, 2011, 2016). On the other hand, we examined the influence of task frequency on modality compatibility effects in task switching while replicating the modality compatibility effect with pedal responses. We also demonstrated that the modality compatibility effect with pedal responses extends to tasks with arbitrary mappings (i.e., without dimensional overlap, Kornblum et al., 1990).

In Experiment 1, we found higher switch costs for modality-incompatible tasks compared to modality-compatible tasks. This finding was replicated in Experiment 2. However, Experiment 2 had two important methodological differences to Experiment 1: First, Experiment 1 used tasks with arbitrary S-R mappings, ruling out that dimensional overlap within tasks is a prerequisite for the influence of modality compatibility on switch costs, and we still found a similar data pattern as in Experiment 1. Second, we included a frequency manipulation, and we found the effect of modality compatibility on switch costs both for frequent tasks and for infrequent tasks. However, in Experiment 2, switch costs were numerically higher for the frequent condition, contrary to our expectation that switch costs for modality-incompatible tasks should further increase for the infrequent condition. We did not observe a modulation of the modality-compatibility effect on switch costs by practice level or the number of successive repetitions of the frequent task, suggesting that the influence of modality compatibility on switch costs is remarkably robust.

Note that we did not find higher RT for modality-incompatible tasks in single-task blocks. Moreover, in Experiment 2 there was even a benefit for incompatible modality pairings in single tasks, strongly indicating that any beneficial effects of modality-compatible tasks in task switching may not be due to pre-experimental differences in task difficulty (for further discussion see also Stephan & Koch, 2010, 2011). In single-task blocks, only one task, and therefore only one combination of stimulus and response modality, has to be held active throughout the block. This suggests that higher switch costs for modality-incompatible tasks arise rather between tasks than within tasks because the two task sets (i.e., the task sets including the two stimulus modalities and the two response modalities) have to be kept active at a time (see also Schacherer & Hazeltine, 2017).

The finding of higher switch costs in modality-incompatible tasks using pedal responses replicates earlier results with manual responses reported by Stephan and Koch (2010, 2011, 2016). Because modality-compatibility effects on switch costs could not be found with oculomotor responses (Stephan et al., 2013), the present finding represents important confirmation of the modality-priming idea underlying the concept of modality compatibility. Specifically, we assume that, in the modality-compatible conditions, the match between the stimulus modality and the modality of the predictable sensory response effects primes responses in the correct modality. In contrast, in the modality-incompatible condition, the response-selection-based activation of the representation that corresponds to the predicted response effect actually refers to the stimulus modality that is assigned to the competing task, hence creating between-task crosstalk in the sense of task confusion. That is, interference arises in modality-incompatible switch trials because the primed response modality has to be inhibited in order to use the incompatibly-mapped response modality instead (for further discussion see also Stephan & Koch, 2016).

With respect to pedal responses, we suggest that pedal responses are more strongly associated with visual stimulation than with auditory stimulation because pedal responses are most often used in the context of locomotion. Therefore, visual stimuli prime the sensory consequences of pedal responses, which facilitates selecting the correct output modality in task switching. The present findings observed in two experiments thus suggest that the influence of modality compatibility in task switching is based on a general principle, and that oculomotor output seems to be an exception, possibly because visual orientation is tied to multiple input modalities that include both visual and auditory stimulation (e.g., Zambarbieri, 2002).

Because we used task sets with dimensional overlap (see Kornblum et al., 1990) in Experiment 1, interference might have increased due to the spatial S-R compatibility between the presented stimulus and the spatially compatible response in both response modalities. However, the replication of the modality-compatibility effect in Experiment 2 using tasks without dimensional overlap supports the assumption that modality-specific crosstalk rather than automatic response activation based on dimensional overlap is responsible for higher switch costs with the modality-incompatible tasks. This finding, using pedal responses in the present experiments, corresponds with the results of Stephan and Koch (2011) and indicates that dimensional overlap within (and across) tasks is not a necessary precondition for the effect of modality compatibility on switch costs.

Apart from generalizing the modality compatibility effect (see also Göthe, Oberauer, & Kliegl, 2016; Hazeltine et al., 2006, for evidence in dual-task experiments), we were interested in possible effects of task frequency on interference in modality-incompatible tasks, namely in the effects of prolonged intervals between switch trials. Note that our research question aimed at the intervals between two executions of the same task and not at the effects of prolonged time between response and stimulus (i.e., RSI), which have already been investigated by Stephan and Koch (2010). They found that switch-cost differences due to modality compatibility dissipate with longer RSI (see also Hazeltine & Ruthruff, 2006, for a similar effect of the stimulus-onset asynchrony in dual-tasking). In the present study, Experiment 2 revealed still higher switch costs for modality-incompatible tasks even when task switches were rare and one of the two tasks was performed only infrequently. This indicates that the occurrence of between-task crosstalk is largely independent of task-switch frequency and interference can even be measured in blocks that differ from single-task blocks only in a few task-switch trials.

We hypothesised further that interference should increase for modality-incompatible *infrequent* tasks with longer intervals between task executions, not only because priming of the frequently used preferred response modality might be harder to overcome after repeated use, but also because the originally less stable task set of the incompatibly paired modalities might suffer from longer gaps between task executions. Our results, showing numerically larger switch costs for modality-incompatible *frequent* tasks, do not support this hypothesis. However, as was already discussed above, we do not find a repetition benefit for the infrequent modality-incompatible task, possibly due to presenting only five repetition trials per block. Thus, differences in RT on repetition trials are presumably responsible for the larger switch costs in frequent modality-incompatible tasks. Deviating results might be obtained with a different ratio of frequent to infrequent tasks or with more trials per block, allowing a higher number of direct repetitions for the infrequent task. However, more research is needed at this point. Nonetheless, we separately replicated the effect of modality compatibility on switch costs for both frequency conditions, showing the robustness of the effect as it is also present in the infrequent task.

The effect of time between task executions was further analysed with respect to switch recency, that is, switch trials for the infrequent task as a function of the number of successive repetitions of the frequent task beforehand. We found an overall effect on RT but no modulation by modality compatibility. This finding leads us to question the hypothesis that task sets of modality-incompatible tasks are subject to greater time-based dissipation than task sets of modality-compatible tasks since we did not find a significant interaction between switch recency and modality compatibility. On the other hand, no priming benefit for modality-compatible infrequent tasks was found that would facilitate selecting the correct response modality after long intervals between task executions.

To conclude, this study showed that the effect of modality compatibility can be generalized to pedal responses. Thus, our results extend findings using manual responses and importantly, demonstrated that modality compatibility is a general principle affecting switch costs in task switching. Finding the modality-compatibility effect when task switches are rare and demonstrating its independence of switch recency proves this effect to be very robust. These results underline the importance of considering the influence of modality compatibility both in theoretical accounts of task switching as well as in applied multitasking settings.

## DATA ACCESSIBILITY STATEMENT

Data is accessible under *https://osf.io/4q6pn/?view_only=9c3f52cfc43d471688f45422d73cacbd*.

## Footnotes

Please note, that nevertheless single-task blocks were included in the present study to control for pre-experimental differences in task difficulty.

Based on the data from Stephan & Koch (2015), where the effect size for the interaction of modality compatibility and switching was medium (η_p_^2^ = .542), a sample size of 8 participants would already have been sufficient to detect such a medium effect with a power of .95 (Faul, Erdfelder, Lang, & Buchner, 2007).

As a manipulation check, we analysed whether the influence of switch frequency increased with practice. To this end, the experimental blocks were split in three sections (i.e., practice level) and this variable was added to the three-way ANOVA reported above. Due to missing values for the infrequent task in one section, 15 (of 16) participants entered this analysis. We report only effects including the new variable. Importantly, the interaction between practice level and frequency was significant, *F*(2,28) = 3.583, *p* < .05, η_p_^2^ = .204. The difference between frequent and infrequent task trials increased with practice (86 ms vs. 129 ms vs. 134 ms). RT in infrequent task trials increased slightly over time, whereas RT in frequent task trials showed no consistent pattern. However, no modulation of the interaction between modality compatibility and transition by practice level was found, *F* < 1, just as all other effects of practice level were non-significant in this exploratory analysis, all *p*s > .08. Because of the relatively low PEs, we did not perform analyses of effects of practice level or lag on PE. Note that a previous study (Schäffner, Koch, & Philipp, 2018) demonstrated that practice in semantic categorization tasks, while leading to general learning benefits, did not influence the effect of modality compatibility on switch costs.
